# Effectiveness of Long Term Supervised and Assisted Physiotherapy in Postsurgery Oral Submucous Fibrosis Patients

**DOI:** 10.1155/2016/6081905

**Published:** 2016-11-03

**Authors:** S. Kale, N. Srivastava, V. Bagga, A. Shetty

**Affiliations:** Department of Oral and Maxillofacial Surgery, Sri Rajiv Gandhi College of Dental Sciences and Hospital, Bangalore, India

## Abstract

Oral submucous fibrosis is one of the leading potentially malignant disorders prevailing in India. A number of conservative and surgical treatment options have been suggested for this potentially malignant disorder (Arakeri and Brennan, 2013). While the role of physiotherapy has been highlighted in the conservative management, its importance in postsurgical cases to avoid scar contracture and subsequent relapse has not been given due importance in the literature. The following is a case report of a male patient surgically treated for OSMF (oral submucous fibrosis) and meticulously followed up for recalls and physiotherapy. The constant supervision and motivation for physiotherapy along with the constant assistance helped achieve satisfying results.

## 1. Introduction

Oral submucous fibrosis (OSMF) is one of the leading potentially malignant disorders prevailing in India. T. Karemore and V. Karemore in their article estimate the number of patients suffering from OSMF in India to be approximately 5 million [1]. The risk of malignant transformation for these is also suggested to be ranging from 7% to 30% [2]. Of the many treatment modalities available for this condition, surgical release of the fibrous bands is one of these. However, treatment outcome relies heavily on patient compliance and cooperation and undeterred dedication towards active physiotherapy postoperatively. Relapses in many of the cases of OSMF treated by surgery have been attributed to insufficient physiotherapy on the part of the patient. This case report attempts to highlight a case of OSMF treated by surgery, followed up postoperatively twice a day for a period of 2 months to oversee and encourage sufficient physiotherapy.

## 2. Case Report

A patient, aged 30 years and a painter by profession, presented to a private clinic with a chief complaint of burning sensation over the cheek on both sides and a noticeable decline in the amount of mouth opening starting 7 years ago. He was advised on mouth opening exercises by the dental practitioner. However, due to the severe pain experienced by the patient during the mouth opening exercises, the patient discontinued the physiotherapy. The patient reported back to the practitioner and was prescribed intralesional injections of hyaluronidase and dexamethasone with strict instructions to abstain from areca nut consumption completely in all forms.

Despite completing the course of intralesional injections bilaterally, the patients complaints did not subside.

On reporting to our department, a thorough clinical examination was performed. The habit history revealed the following habits:

1. A chronic cigarette smoker, smoking 5 cigarettes a day for the past 10 years
2. A chronic gutka chewer, consuming 10 packets of gutka per day, for the past 6 years
3. An occasional alcohol consumer for the past 10 years
4. An occasional pan chewer for the past 6 years

**Figure 1:**
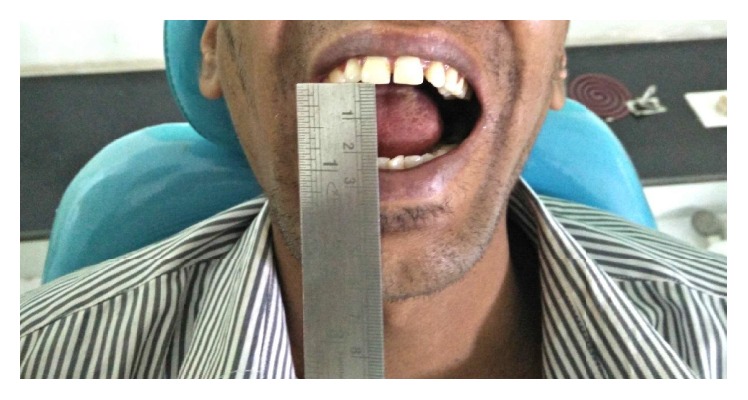
Preoperative MO—22 mm.

Clinical findings revealed the following:

1. A mouth opening of 22 mm (Figure 1)
2. Blanching of the buccal mucosa bilaterally and over the soft palate
3. Erythematous patches over the buccal mucosa
4. Hockey stick shaped uvula
5. Restricted mobility of the tongue, with the tongue on protrusion, slightly overlapping the lower incisors
6. Stains and calculus
7. Palpation that confirmed the inspectory findings and exhibited presence of vertical fibrous bands over the buccal mucosa and horizontal bands circumorally

The previous history of intralesional injections and the minimal benefit from them drove the treatment plan in favour of surgical resection of the fibrous bands bilaterally in the region of the buccal mucosa.

**Figure 2:**
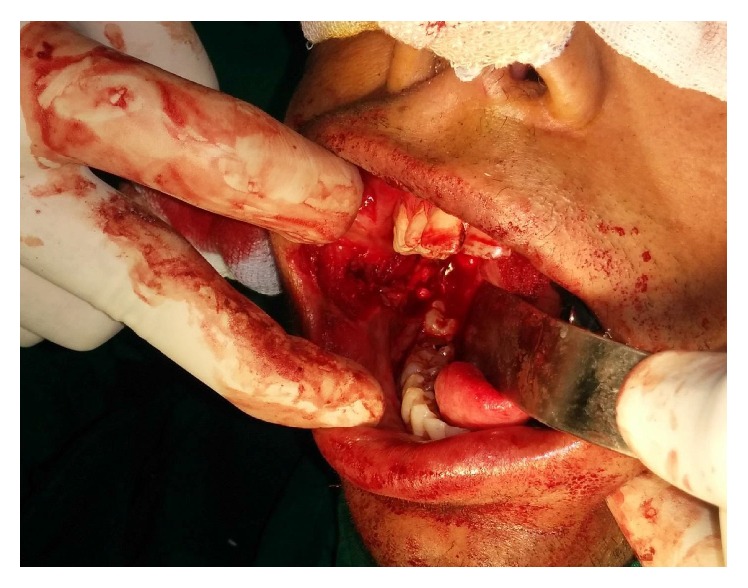
Surgical resection of fibrous bands.

**Figure 3:**
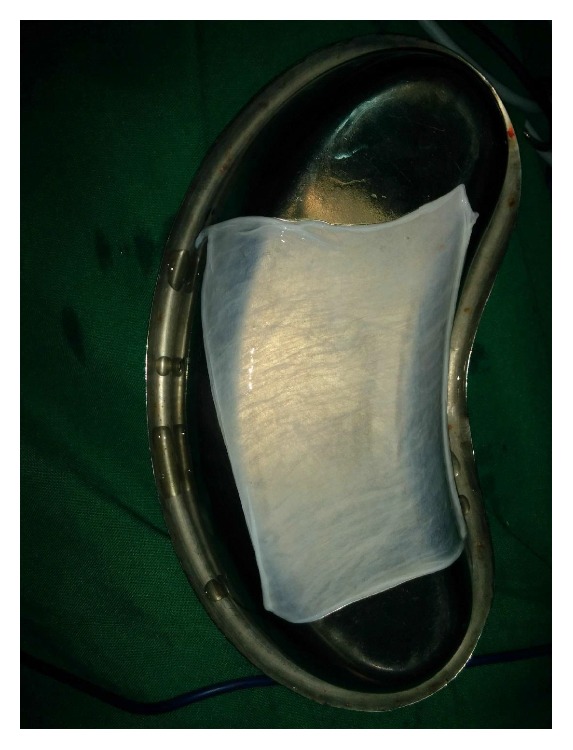
Collagen membrane impregnated with placental extract and hyaluronidase.

**Figure 4:**
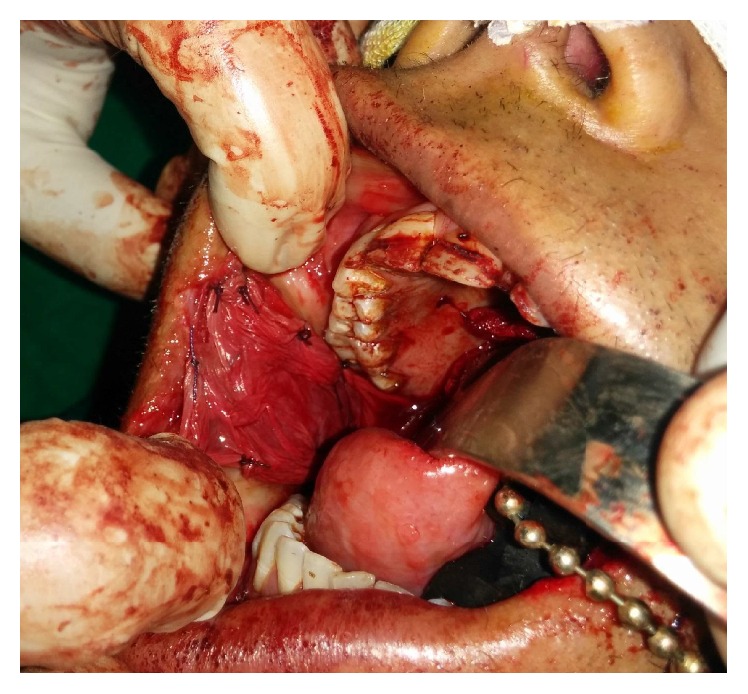
Collagen membrane sutured on the defect over the buccal mucosa.

The preoperative investigations were done and found to be within normal limits. Informed consent for surgery was obtained from the patient. Surgical resection of the fibrous bands was carried out under general anaesthesia (Figure 2). Postresection mouth opening achieved was 32 mm. To further enhance the mouth opening, a plan for bilateral coronoidectomy was decided on the operating table which further improved the mouth opening to 48 mm. The resultant defect over the buccal mucosa was covered with a collagen membrane impregnated with placental extract and hyaluronidase (Figure 3) [3]. The collagen membrane was sutured over the defect (Figure 4) and a bolster gauze was placed over it to stabilize the membrane. A Ryles tube was inserted postoperatively to aid in feeding.

**Figure 5:**
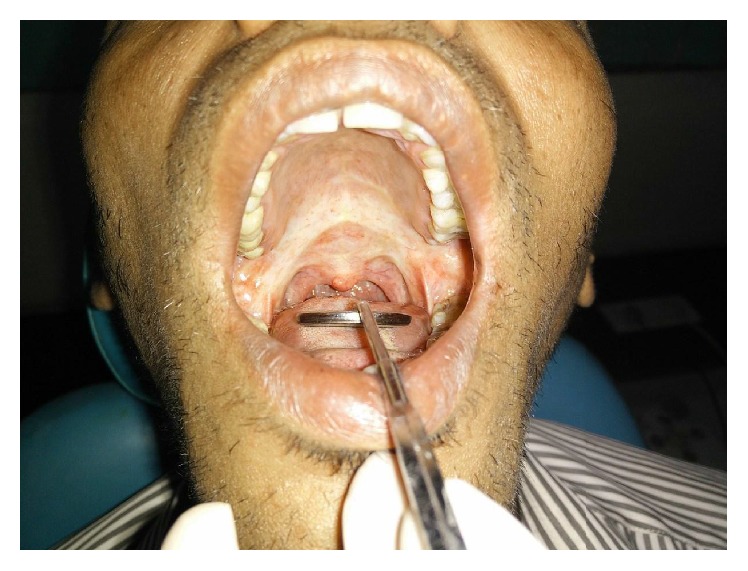
Hockey stick shaped uvula and the blanching of the palate.

Active physiotherapy was started from the 1st postoperative day under the cover of strong analgesics (intramuscular diclofenac sodium 3 mL BD). A Hester's jaw opener was used actively to open the mouth. The patient was discharged on the second postoperative day after a session of assisted physiotherapy. From the third postoperative day onwards, the patient was advised to report to our department every morning for a period of 2 months. Active physiotherapy using Hester's jaw opener was performed in the department. The patient was again attended to everyday in the evenings for a session of active physiotherapy and was also simultaneously encouraged. Targets were set for the patient to attain specific mouth openings till certain days to encourage him. In cases where a complaint of pain incapacitated the patient from doing physiotherapy, the attending surgeons personally helped the patient use the Hester's gradually. The patient was advised to perform physiotherapy himself using the jaw opener between these 2 assisted sessions of physiotherapy. Mouth opening was gradually increased and the wound was evaluated regularly. Wound was evaluated on the 10th postoperative day and certain loose sutures were removed. Betadine irrigation was also done. The characteristic hockey stick shaped uvula and the blanching of the palate associated with OSMF could also be observed clearly after surgery (Figure 5).

**Figure 6:**
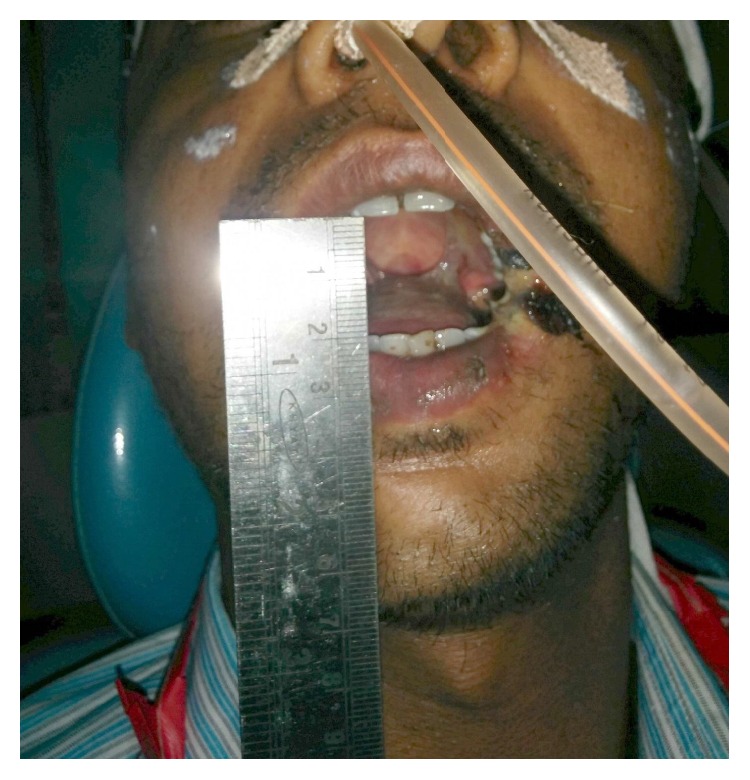
3rd postoperative day* immediately after* assisted mouth opening—21 mm.

**Figure 7:**
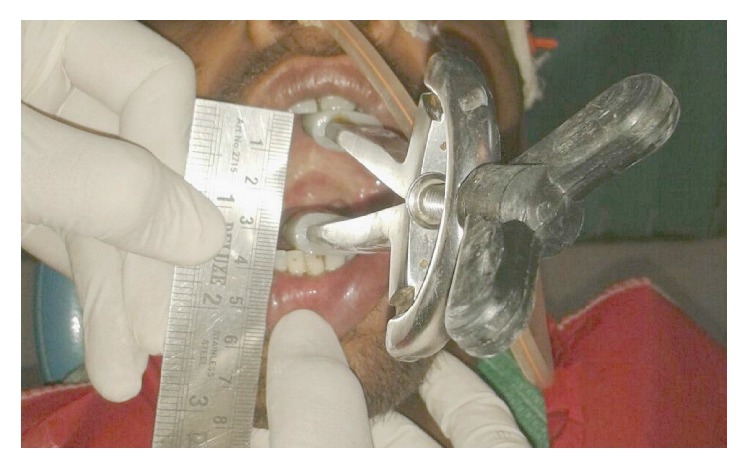
7th postoperative day assisted mouth opening—33 mm.

**Figure 8:**
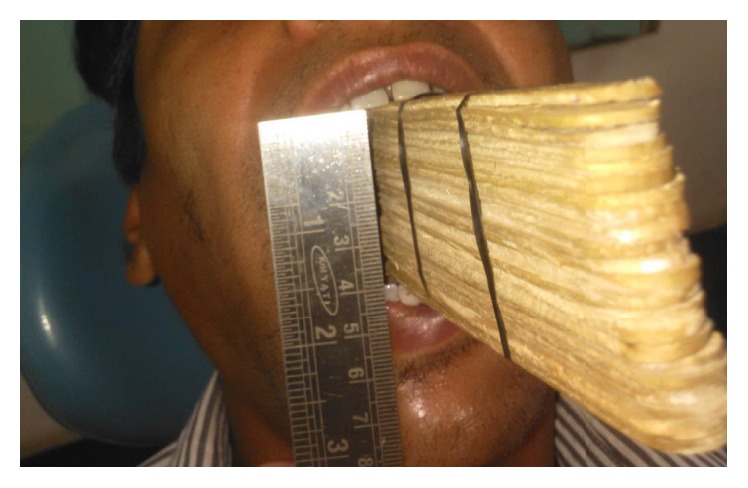
30th postoperative day ice cream sticks assisted mouth opening—40 mm.

**Figure 9:**
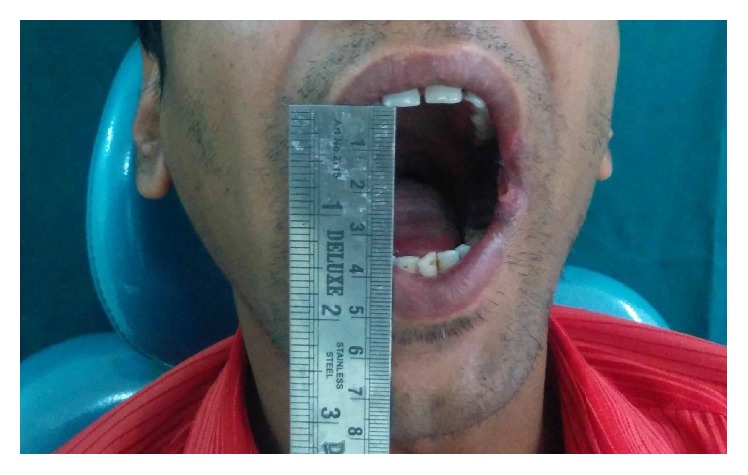
35th postoperative day passive mouth opening—35 mm.

**Figure 10:**
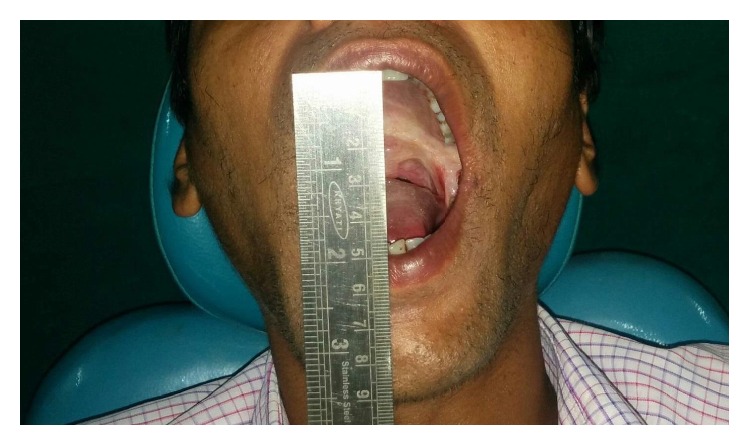
6-month postoperative passive mouth opening—43 mm.

The improvement in mouth opening observed was as follows:

1. 3rd postoperative day assisted mouth opening—21 mm (Figure 6)
2. 7th postoperative day assisted mouth opening—33 mm (Figure 7)
3. 1-month postoperative assisted mouth opening—40 mm (Figure 8)
4. 35-day postoperative passive mouth opening—35 mm (Figure 9)
5. 6-month postoperative passive mouth opening—43 mm (Figure 10)

## 3. Discussion

The association of OSMF with India dates back to the times of Sushruta, who recognized OSMF as a mouth and throat malady and termed it “Vidhari” in around 3000 BC [4]. Schwartz in 1952 first found the existence of this condition in five Indian women from Kenya [5]. Named initially as “atrophia idiopathica (tropica) mucosae oris” by him, it was later renamed “submucous fibrosis” by Joshi in 1953 [5]. Of the many reasons cited for the recurrence of OSMF after surgery or after other treatment modalities, insufficient physiotherapy is one of the major causes. While nonstoppage of the habit can be attributed to the addictive nature of areca nut, insufficient physiotherapy mainly results from the following causes:

1. Negligence and underestimation on part of the patient towards the importance of performing physiotherapy in the right way and for the right number of times
2. Pain during the physiotherapy incapacitating the patient from doing active physiotherapy on his own

A number of studies have been performed to assess the effectiveness of physiotherapy as a conservative treatment modality for mild to moderate cases of OSMF. Thakur et al. [6] in their study on 64 patients observed physiotherapy to be a helpful adjunct to micronutrients for conservative management of patients with mild to moderate OSMF. The results were statistically significant when physiotherapy and micronutrient therapy were used in combination compared to physiotherapy being used alone.

Vijayakumar and Priya [7] in their study on 64 patients with grade 2 and 3 OSMF evaluated the role of physiotherapy and ultrasound therapy in conservative management of such patients. The mean improvement in mouth opening obtained was 6 mm suggesting that heating a muscle and subsequent physiotherapy can help achieve improved mouth opening.

Alam et al. [8] through their study on patients with OSMF advocated physiotherapy postsubmucosal injections of chemicals for treating OSMF. The main objective of the physiotherapy according to the author was to counteract the tendency of fibrosis, trismus, and dysphagia occurring after the trauma due to the injections and the irritative nature of the chemicals injected.

The literature however is scarce on the importance of physiotherapy after surgery to reduce chances of scar contracture and relapse. A study done by Cox and Zoellner on 54 OSMF patients highlighted the importance of physiotherapy in improving mouth opening [9]. Physiotherapy was employed as the sole modality of conservative treatment as compared to the use of local injections of hyaluronidase. The results of the study pointed towards a significant improvement in mouth opening in the physiotherapy group. Many studies invariably mention the need for physiotherapy after surgery to improve outcomes and prevent scar contracture and recurrence [10–12]. However, the initiation of physiotherapy, its importance, and the desirable duration to continue physiotherapy after surgery have not been discussed much. A common observation is the patients aversion towards immediate postoperative physiotherapy due to the associated severe pain. However, we promote starting active physiotherapy immediately 2 days postoperatively to minimize chances of scar contracture setting in. To overcome the pain, we advocate keeping the patient under a strong analgesic cover. Also, another reason for the disappointment noted with postsurgery outcomes is the poor compliance on part of the patient to perform physiotherapy to the desired extent, for the desired frequency and in the correct way. Home programmes of physiotherapy can be successful only if the patient is motivated and made to do the exercises once every day under supervision. Motivating the patient and a constant supervision and assistance towards physiotherapy were hence our main objectives. Without supervision and motivation, patients tend to fall short of the desired goal of mouth opening. Repeated over days, this results in a gradual decline in the potential increase in mouth opening which could have been achieved.

The success towards achieving a satisfactory mouth opening in this patient can be attributed to the active supervised physiotherapy. The patient visited the hospital regularly for a period of 2 months for assisted physiotherapy and was in turn attended to for the same duration by an attending surgeon. This is not feasible for every patient as the routine and compliance will vary for every individual.

The following case report is an attempt to highlight the need for our intervention into the patients' home programmes of physiotherapy to maximize the benefits obtained from these. The results can be more definitively seen in controlled trials as are going on in our institution.
